# Vaccinating Girls and Boys with Different Human Papillomavirus Vaccines: Can It Optimise Population-Level Effectiveness?

**DOI:** 10.1371/journal.pone.0067072

**Published:** 2013-06-26

**Authors:** Mélanie Drolet, Marie-Claude Boily, Nicolas Van de Velde, Eduardo L. Franco, Marc Brisson

**Affiliations:** 1 Centre de recherche du CHU de Québec, Hôpital Saint-Sacrement, Québec, Canada; 2 Département de médecine sociale et préventive, Université Laval, Québec, Canada; 3 Department of Infectious Disease Epidemiology, Imperial College, London, United Kindom; 4 Division of Cancer Epidemiology, McGill University, Montreal, Canada; Instituto Butantan, Brazil

## Abstract

**Background:**

Decision-makers may consider vaccinating girls and boys with different HPV vaccines to benefit from their respective strengths; the quadrivalent (HPV4) prevents anogenital warts (AGW) whilst the bivalent (HPV2) may confer greater cross-protection. We compared, to a girls-only vaccination program with HPV4, the impact of vaccinating: 1) both genders with HPV4, and 2) boys with HPV4 and girls with HPV2.

**Methods:**

We used an individual-based transmission-dynamic model of heterosexual HPV infection and diseases. Our base-case scenario assumed lifelong efficacy of 100% against vaccine types, and 46,29,8,18,6% and 77,43,79,8,0% efficacy against HPV-31,-33,-45,-52,-58 for HPV4 and HPV2, respectively.

**Results:**

Assuming 70% vaccination coverage and lifelong cross-protection, vaccinating boys has little additional benefit on AGW prevention, irrespective of the vaccine used for girls. Furthermore, using HPV4 for boys and HPV2 for girls produces greater incremental reductions in SCC incidence than using HPV4 for both genders (12 vs 7 percentage points). At 50% vaccination coverage, vaccinating boys produces incremental reductions in AGW of 17 percentage points if both genders are vaccinated with HPV4, but increases female incidence by 16 percentage points if girls are switched to HPV2 (heterosexual male incidence is incrementally reduced by 24 percentage points in both scenarios). Higher incremental reductions in SCC incidence are predicted when vaccinating boys with HPV4 and girls with HPV2 versus vaccinating both genders with HPV4 (16 vs 12 percentage points). Results are sensitive to vaccination coverage and the relative duration of protection of the vaccines.

**Conclusion:**

Vaccinating girls with HPV2 and boys with HPV4 can optimize SCC prevention if HPV2 has higher/longer cross-protection, but can increase AGW incidence if vaccination coverage is low among boys.

## Introduction

Infection with human papillomavirus (HPV) types is a necessary cause of cervical cancer, with HPV-16/18 accounting for 70% of these cancers. The other most frequent oncogenic HPV types worldwide (HPV-31,-33,-45,-52, and -58) contribute to an additional 20% of cervical cancers [1]. Infection with high oncogenic risk types, mainly HPV-16, has also been associated with other anogenital (vulvar, vaginal, anal, penile) and head and neck cancers [2]–[4]. Infection with low oncogenic risk types, such as HPV-6 and -11, is associated with anogenital warts (AGW) [5] and recurrent respiratory papillomatoses [6]. Although it is well recognized that HPV causes substantial burden of diseases in women, the burden in men is also considerable [7]. The number of non-cervical HPV-related cancers that occur each year is about the same for men and women [7].

Two prophylactic HPV vaccines are currently licensed for use in females in many countries: the bivalent and quadrivalent vaccines that protect against types HPV-16/18 and HPV-16/18/6/11, respectively. Given evidence that the HPV vaccines are highly efficacious (vaccine efficacy against persistent infections and cervical lesions (VE) = 98–100%) [8], [9], and cost-effective in preadolescent females [10], most developed countries have introduced routine vaccination of girls. Many of these countries use the quadrivalent vaccine (e.g., the U.S., U.K., Canada and Australia) [11]–[14].

A randomized clinical trial has shown the HPV quadrivalent vaccine to be safe and efficacious against persistent infections (VE = 86%) and external genital lesions (VE = 90%) in young males, and against precancerous anal lesions in men-who-have-sex-with-men (MSM) (VE = 78%) [15], [16]. Following these results, the quadrivalent vaccine has been licensed for use in males in several countries [11], [17], [18]. However, only a few countries, including the U.S. and Australia, have introduced male/female HPV immunization programs using the quadrivalent vaccine. In many countries, the main barrier for vaccinating boys is cost-effectiveness. Most studies suggest that vaccinating boys in addition to girls is unlikely to be cost-effective if vaccination uptake is high among girls (i.e. >50%), due to herd immunity effects [19]–[25].

Policy makers examining male/female vaccination programs may consider vaccinating girls and boys with different vaccines to benefit from their respective potential strengths and differential costs. This option has been examined in Quebec, Canada. Evidence suggest that the quadrivalent vaccine prevents AGW in both females and males (herd immunity) following girl-only vaccination programs [26]–[28]. On the other hand, the bivalent vaccine may confer greater cross-protection against high oncogenic risk HPV-types 31/33/45/52/58 [29] and/or longer duration of protection against the vaccine HPV-types 16/18 [30]. Therefore, using the bivalent vaccine for girls and the quadrivalent for boys could potentially increase the population-level effectiveness against cervical lesions and cancer without significantly impacting the effectiveness against AGW (because of herd immunity from vaccinated boys). Hence, using the bivalent and quadrivalent HPV vaccines could represent an opportunity to share the potential advantages and uncertainties of the vaccines described above.

The objective of this study is to examine and compare the potential incremental impact of two male/female HPV vaccination strategies (versus girls-only quadrivalent vaccination): 1) vaccinating both genders with the quadrivalent vaccine, and 2) vaccinating boys with the quadrivalent vaccine and switching girls to the bivalent vaccine.

## Methods

We developed HPV-ADVISE (Agent-based Dynamic model for VaccInation and Screening Evaluation), an individual-based transmission-dynamic model of partnership formation and dissolution, and natural history of multi-type HPV infection and disease [31], [32]. Individuals in the model are attributed three risk factors for HPV infection and disease: gender, a level of sexual activity (Low = L0 to High = L3) and a screening behaviour (No screening = S0 to High screening frequency = S4). Eighteen HPV-types are modeled individually, including the vaccine and cross-protective types. It is assumed that the natural history (e.g., transmission, persistence, disease progression) of a specific HPV-type is independent of co-infections within an individual. The HPV diseases included in the model are AGW, cervical cancer, and cancers of the vulva, vagina, penis, anus, and oropharynx. Vaccine efficacy is type-specific and can be applied to any of the 18 HPV-types included in the model. Each vaccinated individual is given a specific duration of protection against the vaccine types sampled from a normal distribution. Cytology-based cervical cancer screening, which prevails in Canada, was assumed for the models. Screening rates are a function of a woman’s screening behaviour level, previous screening test results, and age.

The sexual behaviour, natural history and cervical screening parameters were identified through calibration (see Van de Velde et al. [32] and http://www.marc-brisson.net/HPVadvise.pdf for methods, parameter values and model fit). We identified 10 parameter sets (out of 285,000) that fit simultaneously 639 pre-specified sexual behaviour, HPV epidemiology and screening data targets [33]–[45]. Variability surrounding model predictions is presented as the median, 10th and 90th percentiles of results from the posterior parameter sets, referred to as the 80 percent range (80%R).

In our base case, we assumed that vaccine efficacy against HPV-vaccine types is 100%, vaccine efficacies against non-vaccine HPV-types are the published type-specific efficacies against persistent infection [29], and vaccine protection (including cross-protection) is lifelong. A lifelong protection was chosen for our base case to illustrate the maximum difference that could be obtained from the two vaccination strategies. However, analysis using shorter durations of protection (20 years for vaccine-types and 10 years for cross-protection [29], [46], [47]) were performed to examine the sensitivity of results to this uncertainty. Sensitivity analyses were also performed by varying vaccination coverage and cross-protective efficacy. Vaccination coverage was varied to represent the situation in different countries (e.g. coverage <50% in the U.S. and >70% in Australia or Canada). We also examined scenarios where the vaccination coverage of boys was lower than in girls, to represent results from acceptability studies of boys’ vaccination among parents [48], [49] and we varied cross-protective efficacy by using different estimates available in the literature [29] (Table S1).

## Results

### High Vaccination Coverage of Girls

#### Anogenital warts

**Figure 1:**
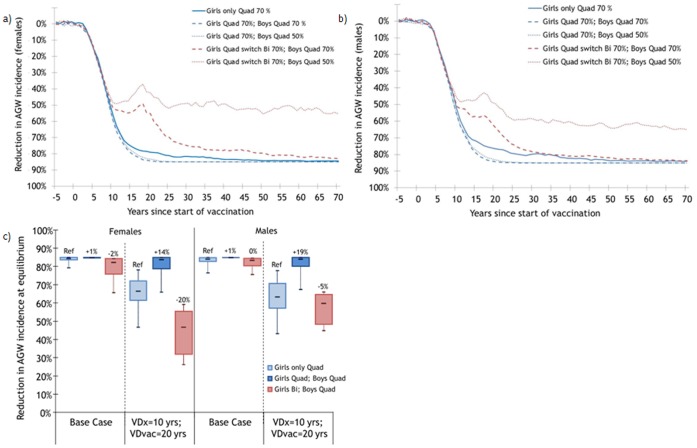
Estimated population-level impact of vaccinating 70% of 12-year-old girls and implementing boys’ vaccination on the incidence of anogential warts. Percentage change following vaccination in a) females and b) males under base assumptions, and c) sensitivity analyses varying vaccine duration and cross-protection. VDx = Vaccine Duration for cross-protective types; VDvac = Vaccine Duration for vaccine types, VEvac = Vaccine Efficacy against vaccine types. Base case: Same characteristics Quad/Bi: VDx = VDvac = lifetime, VEvac = 100%. Sensitivity analyses: Different characteristics Quad/Bi: Quad: VDvac = 20 yrs, VDx = 0 yr, VEvac = 100%; Bi: VDvac = VDx = lifetime, VEvac = 100%. Changes in vaccination strategy occurred 5 years after the beginning of girls-only vaccination. Population of 170,000 individuals. Each parameter set was run 25 times. * the numbers at the top of the boxes represent percentage point changes in the magnitude of the reduction attributable to vaccination. With lifelong duration of the vaccine, HPV6-11 AGW among females and males are eliminated in 45%, 35% and 100% of scenarios when girls only, boys only or both genders are vaccinated with the quadrivalent vaccine (70% coverage). With shorter duration of the vaccine, HPV6-11 AGW are eliminated in 0%, 0%, and 40% of scenarios when girls only, boys only or both genders are vaccinated with the quadrivalent vaccine (70% coverage).

Under base assumptions, the model predicts that vaccinating 70% of 12-year-old girls with the quadrivalent vaccine will produce a rapid decrease in the overall incidence of AGW (Figure 1a–b). At equilibrium (70 years post-vaccination), the incidence of AGW is estimated to be reduced by 84% (80%R:79,85) in females and 84% (80%R:76,85) in heterosexual males. Adding the vaccination of 70% of 12-year-old boys with the quadrivalent vaccine is expected to produce very small incremental reductions in the incidence of AGW, irrespective of whether the girls are switched to the bivalent or remain with the quadrivalent vaccine (Figures 1a–b–c).

In a male/female program, base case results are sensitive to the vaccination coverage achievable among boys. If boys have a lower vaccination coverage than girls (girls:70%, boys:50%), and girls are switched to the bivalent vaccine, the model predicts that the equilibrium incidence of AGW will be 31 percentage points higher (80%R:24,39) in females and 19 percentage points higher (80%R:16,22) in heterosexual males compared to girls-only quadrivalent vaccination (Figures 1a–b). This is because the effective population-level coverage against HPV-6/11 would be reduced from about 35% (girls:70%, boys: 0%) to 25% (girls:0%, boys:50%). Obviously, if both genders are vaccinated with the quadrivalent, high population-level effectiveness against AGW incidence will be maintained even if boys have lower vaccination coverage (Figures 1a–b–c). Under this scenario, the effective coverage against HPV-6/11 would increase from 35% (girls:70%, boys = 0%) when vaccinating girls only to 60% (girls:70%, boys:50%) when vaccinating both genders.

Because the potential to achieve significant gains from vaccinating boys depends on the population-level effectiveness of girls-only vaccination, the incremental benefits of vaccinating boys is particularly sensitive to duration of vaccine protection. When assuming limited duration of protection (20 years) and high coverage (boys & girls = 70%), vaccinating boys in addition to girls with the quadrivalent vaccine produces larger incremental benefits in AGW incidence reduction than when assuming lifelong protection (percentage points: 14 vs. 1 for females and 19 vs. 1 for heterosexual males-Figures 1c). However, when assuming limited duration of protection (20 years), switching girls to the bivalent vaccine leads to important losses in population-level effectiveness against AGW compared to girls-only quadrivalent vaccination (percentage points: −20 for females and −5 for heterosexual males-Figure 1c). This is because, if vaccine duration is shorter, vaccinating only boys against HPV-6/11 does not induce the herd effects necessary to counter balance the loss of protection caused by switching girls to a bivalent vaccine. In other words, vaccinating 50% of boys only against HPV-6/11 produces smaller herd immunity effects than vaccinating 50% of girls only. These results suggest that vaccinating boys does not produce the same level of herd immunity to girls than vice versa. This is most likely because females have male partners that are generally older than them, and the average duration of infection is longer for females.

#### Cervical intraepithelial neoplasia and cancer

**Figure 2:**
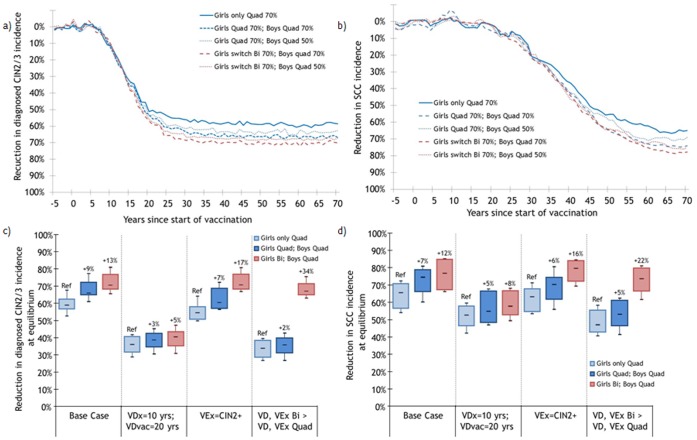
Estimated population-level impact of vaccinating 70% of 12-year-old girls and implementing boys’ vaccination on the incidence of cervical disease. Percentage change following vaccination in the incidence of a) diagnosed cervical intraepithelial neoplasia 2 or 3 (CIN2/3) and b) squamous cell carcinoma (SCC) under base case and impact of sensitivity analyses varying vaccine duration and cross-protection on incidence of c) CIN2/3 and d) SCC. VDx = Vaccine Duration for cross-protective types; VDvac = Vaccine Duration for vaccine types, VEvac = Vaccine Efficacy against vaccine types, VEx = Vaccine Efficacy against cross-protective types. Base case: Same characteristics Quad/Bi: VDx = VDvac = lifetime, VEvac = 100%, VEx = persistent infection (Table S1). Sensitivity analyses: Different characteristics Quad/Bi: Quad: VDvac = 20 yrs, VDx = 0 yr, VEvac = 100% VEx = 0%; Bi: VDvac = VDx = lifetime, VEvac = 100%, VEx = CIN2+ (Table S1). Changes in vaccination strategy occurred 5 years after the beginning of girls-only vaccination. Population of 170,000 individuals. Each parameter set was run 25 times. * the numbers at the top of the boxes represent percentage point changes in the magnitude of the reduction attributable to vaccination. None of the scenarios eliminated CIN2/3 and SCC.

Under base assumptions, the model predicts that vaccinating 70% of 12-years-old girls with the quadrivalent vaccine will reduce the incidence of diagnosed CIN2/3 and SCC by 59% (80%R:53,68) and 66% (80%R:54,72) at equilibrium, respectively (Figures 2a–b). Adding the vaccination of boys with the quadrivalent vaccine (70% vaccination coverage) is expected to produce incremental reductions in CIN2/3 and SCC incidence of 9 (80%R:7,10) and 7 percentage points (80%R:5,11), respectively (Figures 2a–b–c–d). On the other hand, vaccinating boys and switching girls to the bivalent vaccine (70% vaccination coverage for both genders) produces greater incremental reductions in CIN2/3 (13 percentage points, 80%R:10,15) and SCC incidence (12 percentage points, 80%R:8,14) than using the quadrivalent vaccine for both genders (Figures 2a–b–c–d). These greater incremental reductions in cervical disease are attributable to the higher cross-protective efficacy of the bivalent vaccine and the assumption that cross-protection is lifelong.

The difference in incremental benefit in cervical disease prevention between the two male/female vaccination strategies is highly sensitive to vaccine duration and cross-protective vaccine efficacy (Figures 2c–d). When assuming shorter duration of vaccine protection, the incremental benefit of vaccinating boys is limited and the two male/female strategies result in similar incremental gains (Figures 2c–d). This is because, when vaccine duration is limited 1) vaccinating boys does not produce sufficient herd immunity to further reduce CIN2/3 and SCC incidence among females, and 2) cross-protection is too short to produce incremental benefits for the bivalent compared to the quadrivalent vaccine. On the other hand, when assuming greater cross-protection and/or longer duration of protection for the bivalent vaccine, the incremental benefits of vaccinating boys with the quadrivalent and girls with the bivalent are much higher than vaccinating both genders with the quadrivalent vaccine.

### Low Vaccination Coverage of Girls

#### Anogenital warts

**Figure 3:**
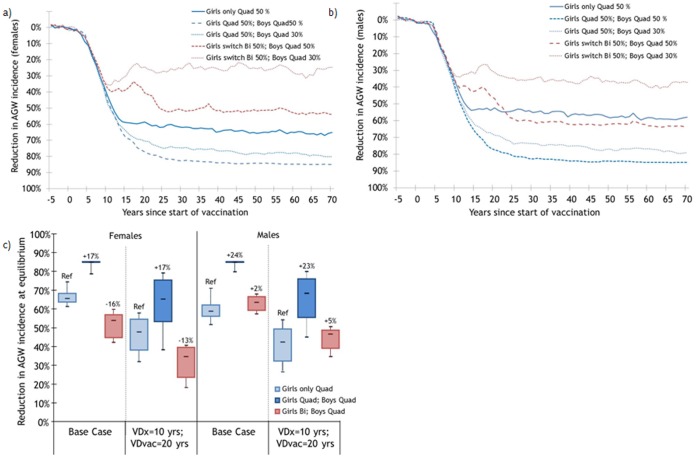
Estimated population-level impact of vaccinating 50% of 12-year-old girls and implementing boys’ vaccination on the incidence of anogential warts. Percentage change following vaccination in a) females and b) males under base assumptions, and c) sensitivity analyses varying vaccine duration and cross-protection. VDx = Vaccine Duration for cross-protective types; VDvac = Vaccine Duration for vaccine types, VEvac = Vaccine Efficacy against vaccine types. Base case: Same characteristics Quad/Bi: VDx = VDvac = lifetime, VEvac = 100%. Sensitivity analyses: Different characteristics Quad/Bi: Quad: VDvac = 20 yrs, VDx = 0 yr, VEvac = 100%; Bi: VDvac = VDx = lifetime, VEvac = 100%. Changes in vaccination strategy occurred 5 years after the beginning of girls-only vaccination. Population of 170,000 individuals. Each parameter set was run 25 times. * the numbers at the top of the boxes represent percentage point changes in the magnitude of the reduction attributable to vaccination. With lifelong duration of the vaccine, HPV6-11 AGW among females and males are eliminated in 0%, 0% and 70% of scenarios when girls only, boys only or both genders are vaccinated with the quadrivalent vaccine (50% coverage). With shorter duration of the vaccine, none of the scenarios eliminated HPV6-11 AGW (50% coverage).

Under base assumptions, the model predicts that vaccinating 50% of 12-year-old girls with the quadrivalent vaccine will reduce AGW incidence by 66% (80%R:61,74) in females and 59% (80%R:52,71) in heterosexuals males at equilibrium (Figures 3a–b). Vaccinating 50% of 12-year-old boys with the quadrivalent vaccine, in addition to girls, is expected to produce incremental reductions in AGW incidence of 17 percentage points (80%R:11,21) in females and 24 percentage points (80%R:14,29) in heterosexual males (Figure 3a–b–c). Conversely, vaccinating boys with the quadrivalent vaccine (50% vaccination coverage) and switching girls to the bivalent vaccine is expected to produce an increase of 16 percentage points (80%R:7,21) in AGW incidence in females whilst maintaining similar level of effectiveness in heterosexual males than the girls-only strategy (Figure 3a–b–c).

If vaccination coverage among boys with the quadrivalent vaccine reaches only 30% and girls are switched to the bivalent vaccine, important increases in the incidence of AGW are expected among both females (36 percentage points,80%R:23,42) and heterosexual males (16 percentage points, 80%R:7,25) (versus girls-only quadrivalent vaccination with 50% vaccination coverage) (Figures 3a–b). In contrast to high coverage, at low vaccination coverage, the duration of vaccine protection has little influence on differences in the incremental gains and losses of the two male/female strategies (Figure 3c).

### Cervical Intraepithelial Neoplasia and Cancer

**Figure 4:**
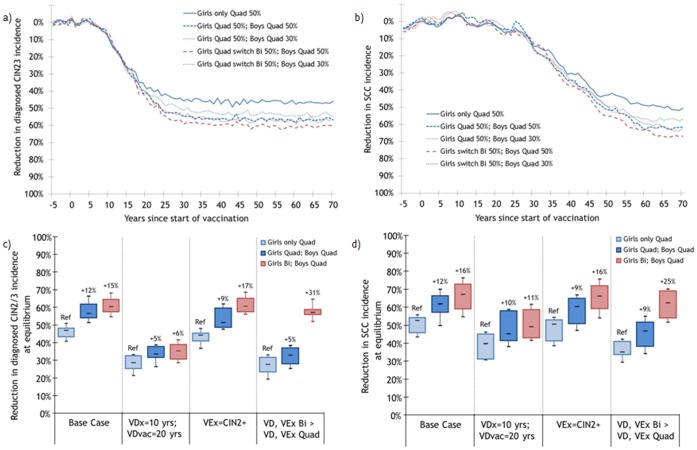
Estimated population-level impact of vaccinating 50% of 12-year-old girls and implementing boys’ vaccination on the incidence of cervical disease. Percentage change following vaccination in the incidence of a) diagnosed cervical intraepithelial neoplasia 2 or 3 (CIN2/3) and b) squamous cell carcinoma (SCC) under base case and impact of sensitivity analyses varying vaccine duration and cross-protection on incidence of c) CIN2/3 and d) SCC. VDx = Vaccine Duration for cross-protective types; VDvac = Vaccine Duration for vaccine types, VEvac = Vaccine Efficacy against vaccine types, VEx = Vaccine Efficacy against cross-protective types. Base case: Same characteristics Quad/Bi: VDx = VDvac = lifetime, VEvac = 100%, VEx = persistent infection (Table S1). Sensitivity analyses: Different characteristics Quad/Bi: Quad: VDvac = 20 yrs, VDx = 0 yr, VEvac = 100% VEx = 0%; Bi: VDvac = VDx = lifetime, VEvac = 100%, VEx = CIN2+ (Table S1). Changes in vaccination strategy occurred 5 years after the beginning of girls-only vaccination. Population of 170,000 individuals. Each parameter set was run 25 times. * the numbers at the top of the boxes represent percentage point changes in the magnitude of the reduction attributable to vaccination. None of the scenarios eliminated CIN2/3 and SCC.

Under base assumptions, the model predicts that vaccinating 50% of 12-years-old girls with the quadrivalent vaccine will reduce the incidence of diagnosed CIN2/3 and SCC by 47% (80%R:41,51) and 53% (80%R:44,56) at equilibrium, respectively (Figures 4a–b). Vaccinating 50% of boys, in addition to girls, with the quadrivalent vaccine is predicted to produce incremental reductions in CIN2/3 and SCC of 12 percentage points (80%R:8,15) and 12 percentage points (80%R:5,16), respectively (Figures 4a–b–c–d). Given the higher bivalent cross-protection, slightly higher incremental reductions in CIN2/3 (15 percentage points, 80%R:12,18) and SCC (16 percentage points,80%R:8,21) incidence are predicted when vaccinating 50% of boys with the quadrivalent vaccine and switching girls to the bivalent vaccine (Figures 4a–b–c–d).

The incremental reductions in CIN2/3 and SCC incidence expected with these male/female vaccination strategies are highly sensitive to vaccine duration and efficacy against cross-protective HPV types (Figure 4c–d). Similarly to scenarios with high vaccination coverage, the two strategies result in very similar incremental reductions in CIN2/3 and SCC incidence if vaccine duration is short (vaccine-type duration = 20 years, cross-protection = 10 years). On the other hand, if the bivalent is assumed to have greater cross-protection and longer duration of protection, switching girls to the bivalent vaccine and vaccinating boys with the quadrivalent is predicted to be the optimal male/female strategy in terms of incremental population-level effectiveness against cervical lesions and cancer (Figure 4c–d).

## Discussion

Our modeling analysis indicates that, at high vaccination coverage of girls (≥70%), adding boys to girls-only quadrivalent vaccination programs will produce very small incremental reductions in AGW incidence. On the other hand, at low vaccination coverage of girls (≤50%), a male/female strategy can substantially improve the prevention of AGW for both, females and males. Both strategies (vaccinating both genders with the quadrivalent vaccine or vaccinating boys with the quadrivalent vaccine and switching girls to the bivalent vaccine) are likely to improve the population-level effectiveness of HPV vaccination against cervical lesions and cancer. Vaccinating girls with the bivalent while vaccinating boys with the quadrivalent vaccine may optimize cervical lesions and cancer prevention, if duration of cross-protection is long. However, to be more broadly successful, this strategy requires achieving sufficiently high coverage of boys with the quadrivalent vaccine to maintain AGW protection among girls through herd immunity. Conversely, if duration of cross-protection is limited, both male/female strategies result in similar incremental effectiveness against cervical lesions and cancers.

If a male/female HPV immunization is implemented, vaccinating girls with the bivalent vaccine and boys with the quadrivalent vaccine could maximize the prevention of cervical lesions and cancers. However, many important uncertainties remain which could threaten the potential benefits of this strategy. Firstly, this strategy requires high vaccination coverage among boys to maintain the level of AGW prevention among girls. However, studies indicate that vaccination coverage may be lower for boys than girls [48], [49]. For example, 67% of mothers intended to have their daughter vaccinated compared to 39% for their son [49] and a preference to vaccinate females over males was observed in several studies [48]. Secondly, our results and those of others [50] suggest that vaccinating boys produces lower levels of herd immunity compared to vaccinating girls. This is most likely because girls have older sexual partners and longer durations of HPV infection [50]. For example when assuming 20 years of protection against HPV-6/11, our model predicts that vaccinating 70% of girls with the quadrivalent will reduce AGW incidence by 63% among heterosexual males, through herd immunity. However, vaccinating 70% of boys will reduce AGW incidence among females by only 47% through herd immunity (Figure 1c). These observations suggest that, even at high coverage, girls could experience substantial rebound in AGW incidence if they are switched to the bivalent vaccine and duration of protection is short. Thirdly, the additional benefit in the prevention of cervical lesions and cancer using the bivalent vaccine (versus the quadrivalent) is highly dependent on the duration of cross-protection. The bivalent vaccine has been shown to be more efficacious against HPV-31/33/45 related infection and cervical lesions, but this efficacy may wane with time [29]. If these preliminary observations are confirmed in future trials, the benefits of switching girls to the bivalent vaccine would be limited. Additional important drawbacks of having two different vaccines are logistical and political. First, having different vaccines for girls and boys can be a logistical challenge, and ultimately could incur greater costs than a one-vaccine strategy. Secondly, depending on public opinion and parental beliefs, having a vaccine that has different properties for girls and boys can lead to discontent with parents of children of one sex (e.g., girls) asking for the vaccine given to the other sex to receive its additional benefits (e.g., quadrivalent for AGW protection).

This is the first study to assess the population-level impact of male/female vaccination with different HPV vaccines for girls and boys. Our results are similar to those examining the incremental benefit of vaccinating boys using the quadrivalent for both genders. Studies have shown limited incremental benefits of vaccinating both genders with the quadrivalent vaccine compared to girls-only vaccination programs [25], [50], and cost-effectiveness studies have consistently reported that this strategy was unlikely to be cost-effective, at high vaccination coverage of girls due to herd immunity [19]–[24]. Evidence suggest that herd immunity could have occurred in Australia and the U.S., where AGW incidence declined in females and heterosexual males [26]–[28], [51] shortly after the introduction of girls-only vaccination programs. Studies have also shown that substantial incremental benefits can be achieved by vaccinating boys when coverage in girls is low [19], [23], [25], [50], [52]. The cost-effectiveness of vaccination programs using different HPV vaccines for girls and boys has yet to be examined. Based on these results, the cost-effectiveness will depend on the relative durations of protection of the HPV vaccines, and on whether the vaccination coverage of boys will be high enough to maintain the effectiveness of girls-only vaccination against AGW (in addition to the cost of the vaccines).

The following considerations should be taken into account when interpreting our results. Firstly, only heterosexual transmission was included in our HPV model. Given that the probability of transmission of HPV is high and that the population of men-who-have-sex-with-men (MSM) is estimated to be small (3–5% in the U.S, Australia and Canada) [33], [53], [54], MSM are unlikely to influence overall HPV transmission at the population-level. Furthermore, although the inclusion of MSM could have slightly increased the benefits of vaccinating boys, it is very unlikely that their inclusion would change our conclusions. Secondly, we purposely decided not to present the incremental effectiveness of the two male/female strategies on the other HPV-related cancers. We have previously shown that there is very little difference between the two HPV vaccines in the population-level effectiveness against these cancers since HPV-16/18 is found in more than 90% of these HPV-positive cancers [31], [32]. Consequently, the incremental benefits of the two strategies will be very similar. Thirdly, although we performed a systematic literature review to identify the most comparable estimates of cross-protection for the two HPV vaccines [29], these estimates were derived from clinical trials with different populations and designs [8], [55], [56], which could partly explain difference in cross-protection between the two vaccines.

The decision to include boys in HPV vaccination programs in the U.S and Australia were partly based on cost-effectiveness [57]. In the U.S, where vaccination coverage is low (44% for at least one dose), adding the vaccination of boys could increase the prevention of HPV-related diseases and be cost-effective. On the other hand, in Australia, where the vaccination coverage of girls is >70%, reduction in vaccine price is thought to have played a determining role in their recommendations. Policy makers in these countries, and others examining whether to include boys in HPV vaccination programs, may now consider using different vaccines for boys and girls to benefit from their respective strengths and share uncertainties/risks between the two genders. Future studies should investigate the cost-effectiveness and acceptability of switching girls to the bivalent vaccine within a male/female program, particularly when considering the risk of reducing the prevention of AGW for both, men and women. In conclusion, vaccinating girls with the bivalent vaccine and boys with the quadrivalent vaccine may optimize population-level effectiveness of HPV vaccination -especially against cancers– under very specific conditions and could increase the burden of AGW under a wide range of realistic scenarios. Given uncertainties/risks regarding the vaccination coverage that can be achieved in boys and the duration of cross-protection of the bivalent vaccine, this strategy should be considered only if vaccination coverage reaches high enough levels in boys to maintain the gains in AGW prevention.

## Supporting Information

Table S1 **Vaccine efficacy (VE) against non-vaccine HPV-types in HPV-naïve females.**
(DOCX)
