# Supplementary material for: Vaccinating Girls and Boys with Different Human Papillomavirus Vaccines: Can It Optimise Population-Level Effectiveness?
Source: PLoS One. 2013 Jun 26;8(6):e67072. doi: 10.1371/journal.pone.0067072 (PMC3694081; doi:10.1371/journal.pone.0067072)
Supplement: Table S1 — Vaccine efficacy (VE) against non-vaccine HPV-types in HPV-naïve females. (DOCX) [file pone.0067072.s001.docx]

**Table S1.** Vaccine efficacy (VE) against non-vaccine HPV-types in HPV-naïve females

|  | Base case | | Sensitivity analyses | |
| --- | --- | --- | --- | --- |
|  | VE persistent infection (%) | | VE CIN2+ (excluding lesions co- infected with HPV-16/18) (%) | |
| HPV-type | Bivalent | Quadrivalent | Bivalent | Quadrivalent |
| 31 | 77.1 | 46.2 | 83.4 | 57.4 |
| 33 | 43.1 | 28.7 | 76.3 | 0.0 ^b^ |
| 45 | 79.0 | 7.8 | 100.0 | 0.0 ^c^ |
| 52 | 8.3 ^a^ | 18.4 | 0.0^d^ | 0.0 ^b^ |
| 58 | 0.0 ^ab^ | 5.5 | 0.0^d^ | 0.0 ^b^ |
| Other HR-types^e^ | 0.0^f^ | 0.0^f^ | 0.0^f^ | 0.0^f^ |

^a^ Data from end of study ATP-E (according-to-protocol cohort for efficacy) cohort, not naïve cohort.

^b^ We used 0 in the model for negative VE estimates.

^c^ There were zero cases in the control group.

^d^ Assumed to be 0%. VE against CIN2+ excluding co-infected lesions with HPV-16/18 are not in the published literature.

^e^ Other High oncogenic risk (HR)-types: 35, 39, 51, 56, 59, 66, 68, 73, 82.

^f^ Assumed to be 0%.
